# Contribution of Functional Antimalarial Immunity to Measures of Parasite Clearance in Therapeutic Efficacy Studies of Artemisinin Derivatives

**DOI:** 10.1093/infdis/jiz247

**Published:** 2019-05-10

**Authors:** Katherine O’Flaherty, Ricardo Ataíde, Sophie G Zaloumis, Elizabeth A Ashley, Rosanna Powell, Gaoqian Feng, Linda Reiling, Arjen M Dondorp, Nicholas P Day, Mehul Dhorda, Rick M Fairhurst, Pharath Lim, Chanaki Amaratunga, Sasithon Pukrittayakamee, Tran Tinh Hien, Ye Htut, Mayfong Mayxay, M Abul Faiz, James G Beeson, Francois Nosten, Julie A Simpson, Nicholas J White, Freya J I Fowkes

**Affiliations:** 1 Burnet Institute, Melbourne, Australia; 2 Centre for Epidemiology and Biostatistics, Melbourne School of Population and Global Health, Melbourne, Australia; 3 Department of Medicine, University of Melbourne, Melbourne, Australia; 4 Department of Immunology, Monash University, Melbourne, Australia; 5 Department of Microbiology, Monash University, Melbourne, Australia; 6 Central Clinical School, Monash University, Melbourne, Australia; 7 Department of Infectious Diseases, Monash University, Melbourne, Australia; 8 Department of Epidemiology and Preventive Medicine, Monash University, Melbourne, Australia; 9 Mahidol-Oxford Tropical Medicine Research Unit, Mahidol University, Bangkok; 10 Faculty of Tropical Medicine, Mahidol University, Bangkok; 11 Shoklo Malaria Research Unit, Mae Sot, Thailand; 12 Centre for Tropical Medicine and Global Health, University of Oxford, United Kingdom; 13 Worldwide Antimalarial Resistance Network, University of Oxford, United Kingdom; 14 Howard Hughes Medical Institute, Chevy Chase, Baltimore; 15 Center for Vaccine Development and Global Health, University of Maryland School of Medicine, Baltimore; 16 Laboratory of Malaria and Vector Research, National Institute of Allergy and Infectious Diseases, National Institutes of Health, Rockville, Maryland; 17 Oxford University Clinical Research Unit, Hospital for Tropical Diseases, Ho Chi Minh City, Vietnam; 18 Department of Medical Research, Yangon, Myanmar; 19 Lao-Oxford-Mahosot Hospital Wellcome Trust Research Unit, Mahosot Hospital, Lao People’s Democratic Republic; 20 Faculty of Postgraduate Studies, University of Health Sciences, Vientiane, Lao People’s Democratic Republic; 21 Malaria Research Group, Chittagong, Bangladesh; 22 Dev Care Foundation, Chittagong, Bangladesh

**Keywords:** Malaria, immunity, antibody, artemisinin, drug resistance

## Abstract

**Background:**

Antibodies to the blood stages of malaria parasites enhance parasite clearance and antimalarial efficacy. The antibody subclass and functions that contribute to parasite clearance during antimalarial treatment and their relationship to malaria transmission intensity have not been characterized.

**Methods:**

Levels of immunoglobulin G (IgG) subclasses and C1q fixation in response to *Plasmodium falciparum* merozoite antigens (erythrocyte-binding antigen [EBA] 175RIII-V, merozoite surface protein 2 [MSP-2], and MSP-142) and opsonic phagocytosis of merozoites were measured in a multinational trial assessing the efficacy of artesunate therapy across 11 Southeast Asian sites. Regression analyses assessed the effects of antibody seropositivity on the parasite clearance half-life (PC_½_), having a PC_½_ of ≥5 hours, and having parasitemia 3 days after treatment.

**Results:**

IgG3, followed by IgG1, was the predominant IgG subclass detected (seroprevalence range, 5%–35% for IgG1 and 27%–41% for IgG3), varied across study sites, and was lowest in study sites with the lowest transmission intensity and slowest mean PC_½_. IgG3, C1q fixation, and opsonic-phagocytosis seropositivity were associated with a faster PC_½_ (range of the mean reduction in PC_½_, 0.47–1.16 hours; *P* range, .001–.03) and a reduced odds of having a PC_½_ of ≥5 hours and having parasitemia 3 days after treatment.

**Conclusions:**

The prevalence of IgG3, complement-fixing antibodies, and merozoite phagocytosis vary according to transmission intensity, are associated with faster parasite clearance, and may be sensitive surrogates of an augmented clearance capacity of infected erythrocytes. Determining the functional immune mechanisms associated with parasite clearance will improve characterization of artemisinin resistance.

## Teaser

Antimalarial immunoglobulin G3 antibodies, complement fixation, and opsonic phagocytosis of merozoites are correlated with parasite clearance rates in therapeutic efficacy studies of artemisinins. By characterizing their contribution across regions of different malaria transmission intensities, artemisinin resistance phenotypes may be more accurately identified.

Resistance to the current first-line antimalarial treatments, the artemisinin derivatives, is now firmly established throughout the Greater Mekong Subregion [1]. Artemisinin resistance manifests as the loss of susceptibility among *Plasmodium falciparum* ring-stage parasites [2, 3]. This presents phenotypically as the slowing of parasite clearance following treatment with artemisinin derivatives [3, 4]. In therapeutic assessments, delayed parasite clearance is defined by either a parasite clearance half-life (PC_½_) of ≥5 hours or, more imprecisely, by persistent parasitemia, confirmed by detection of parasites via microscopy on day 3 after treatment [5]. The slow-clearance phenotype is associated with single-nucleotide polymorphisms within the propeller region of the gene encoding kelch, located on chromosome 13 of *P. falciparum* (*kelch13*) [5, 6]. Not all mutations confer the delayed-clearance phenotype. Both the phenotype and genotype are used in the monitoring of artemisinin resistance in therapeutic efficacy studies [5]. However, characteristics of parasite clearance vary widely in patients with and those without *kelch13* mutations and can be influenced by parasite factors, such as developmental stage [7], and host factors, such as naturally acquired immunity [8, 9], which can affect estimates of artemisinin treatment efficacy in populations.

Naturally acquired immunity develops after repeated exposures to *P. falciparum* [10]. Antibody-mediated immunity limits parasite replication through the opsonization and neutralization of merozoite surface antigens and the fixation of complement factors, thereby preventing erythrocyte invasion [11–13], and contributes to parasite clearance via the opsonization of infected erythrocytes to enhance their phagocytosis and lysis [11, 14, 15]. These functions are mediated predominantly by the cytophilic subclasses of immunoglobulin G (IgG), IgG1 and IgG3 [15–19], which bind with high affinity to Fc receptors on effector cells and possess specific residues on their Fc portion, allowing enhanced complement fixation [20]. The polarization of the malaria parasite–specific IgG1/IgG3 response has been described as dependent on the antigen and the characteristics of exposure (ie, age and transmission intensity) [18, 19, 21–23]. Several cohort studies assessing antimalarial IgG have shown that cytophilic subclasses specific for merozoite antigens, particularly IgG3, are associated with protection against high-density parasitemia and the amelioration of the clinical symptoms of malaria [11, 15–18]. The cytophilic IgG subclasses and associated mechanisms, such as opsonic phagocytosis and complement fixation, may therefore influence the current measures of parasite clearance used in therapeutic efficacy studies, both by targeting the variant surface antigens (VSAs) of infected erythrocytes not killed by treatment and, to a lesser extent, by clearing merozoite stages through opsonization of conserved antigens before they can invade the erythrocyte and mature.

Previous single-site therapeutic efficacy studies of former first-line antimalarials have shown that individuals with higher levels of IgG specific for *P. falciparum* blood stages have a reduced risk of antimalarial treatment failure [8]. However, the relative effect of the cytophilic IgG subclasses and their associated functions on measures of artemisinin treatment efficacy and how this effect varies according to transmission intensity has not been quantified. In a recent multinational therapeutic efficacy assessment of artesunate, we found that total IgG responses targeting the blood stages of *P. falciparum* varied according to transmission intensity and were associated with faster parasite clearance, including in regions with emerging artemisinin-resistant *P. falciparum* [9]. Elucidating the mechanisms involved in mediating parasite clearance will advance our understanding of phenotypic measures of artemisinin resistance. Here, we sought to elucidate the antibody-mediated mechanisms associated with measures of parasite clearance (and treatment efficacy) during artesunate treatment and how they vary by transmission intensity, by measuring levels of the cytophilic IgG subclasses, complement fixation, and opsonic phagocytosis directed against the relatively conserved merozoite stage.

## METHODS

### Study Design and Procedures

Plasma samples were acquired from 984 patients participating in the Tracking Resistance to Artemisinin Collaboration (TRAC) study, a multicenter, randomized, controlled, drug-efficacy trial described in detail previously [5]. Briefly, samples were collected from 11 study sites across 6 countries (Bangladesh, Cambodia [4 sites], the Lao People’s Democratic Republic, Myanmar, Thailand [3 sites], and Vietnam; Table 1). Participants were aged between 6 months and 65 years, were symptomatic with fever or history of fever, and had uncomplicated falciparum malaria diagnosed by light microscopy. Participants were randomly assigned to receive 3-day artesunate monotherapy of 2 or 4 mg/kg, followed by a full course of artemisinin-based combination therapy. Participants from Srisaket, Thailand, and Pursat and Pailin, western Cambodia, all received a 4-mg/kg dose of artesunate, owing to established artemisinin resistance. Patients were hospitalized for at least the first 3 days of treatment, and the presence of parasitemia was determined at 0, 4, 6, 8, and 12 hours and then every 6 hours until parasitemia could not be identified by microscopy on 2 consecutive blood slides. Additional follow-up evaluations were performed on days 7 and 14 at all study sites. The full sequence of *kelch13* was determined for isolates collected at admission, as described previously [5]. All participants or parents/guardians provided informed consent, and ethical approval was provided by the relevant local ethics committees; the Oxford Tropical Research Ethics Committee, United Kingdom (OxTREC 06 11); and the Alfred Hospital Committee for Ethics, Australia (485/12).

**Table 1.** Characteristics of Study Participants

		Age, y			Parasite Density, Parasites/μL		PC_½_, h				
Country, Study Site	Participants, No.	Median (IQR)	Range	Male Sex, %	Median (IQR)	Range	Median (IQR)	Range	PC_½_ ≥ 5 h, Participants, % (Proportion)	Parasitemia at d 3, Participants, % (Proportion)	*kelch13* Mutant, Participants, % (Proportion)
Bangladesh											
Ramu	49	26 (20–35)	10–55	86 (42/49)	32 154 (19 594–50 868,)	10 048–224 196	2.60 (2–3.2)	0.7–5.4	2 (1/49)	2 (1/48)	0 (0/49)
Cambodia											
Pursat	120	25 (19–33)	3–60	91 (109/120)	56 583 (35 670–107 576)	9797–284 861	5.60 (4.3–6.7)	1.7–11.8	61 (73/119)	71 (85/119)	66 (76/115)
Preah Vihear	120	20 (14–29)	4–58	68 (82/120)	56 583 (42 704–86 162)	13 942–311 237	3 (2.5–4.2)	1.2–12.6	22 (26/120)	24 (29/120)	19 (22/113)
Ratanikiri	120	14 (9–19.5)	2–55	65 (78/120)	62 109 (32 844–94 702)	5024–310 860	3 (2.3–3.5)	0.7–8.8	6 (7/120)	9 (11/118)	3 (4/116)
Pailin	99	25 (19–38)	10–57	87 (86/99)	45 216 (25 748–87 543)	3712–389 988	6.10 (4.9–7.2)	2.4–9	74 (71/96)	73 (71/97)	80 (79/99)
Laos											
Attapeu	93	23 (14–29)	6–60	69 (64/93)	50 240 (27 255–90 432)	10 048–198 574	2 (1.6–2.7)	1.1–9.2	6 (5/84)	11 (10/92)	3 (3/92)
Myanmar											
Shwe Kyin	79	24 (19–31)	1–54	82 (65/79)	66 066 (27 088–115 552)	10 640–420 006	3.10 (2.6–4.1)	1.3–8.6	13 (10/77)	15 (12/78)	22 (17/77)
Thailand											
Mae Sot	120	29 (22.5–37)	18–58	78 (94/120)	37 178 (17 584–83 273)	2560–327 062	4.90 (3.7–6.4)	0.6–10.1	50 (58/117)	45 (53/118)	51 (60/117)
Srisaket	41	29 (22–38)	16–54	100 (41/41)	31 902 (13 062–75 360)	4346–192 997	6.95 (4.3–8.7)	1.6–13.9	67 (24/36)	68 (23/34)	83 (30/36)
Ranong	23	33 (26–39)	19–53	70 (16/23)	40 192 (24 618-80 384)	5903-94 451	5.30 (3.5–6.4)	2.4–13.8	59 (13/22)	60 (12/20)	65 (13/20)
Vietnam											
Binh Phuoc	120	26 (18.5–38.5)	3–61	77 (92/120)	49 738 (23 864–96 084)	9797–205 230	3.10 (1.9–5.3)	0.7–8.9	28 (33/118)	32 (38/118)	24 (28/117)

Abbreviations: IQR, interquartile range; PC_½_, parasite clearance half-life.

### Recombinant Antigens

To assess previous *P. falciparum* exposure, we included the preerythrocytic antigen circumsporozoite protein (CSP) as described previously [9]. We selected 3 merozoite-stage antigens that have been shown to illicit protective antibody responses and highly cytophilic IgG responses [17, 18]: merozoite surface protein 2 (full length; MSP-2) [18], erythrocyte-binding antigen 175 regions III–V (amino acid residues 761–1271; EBA-175) [17], and MSP1–42 (amino acid residues 1362–1720). EBA-175 and MSP-2 have been shown previously to mediate a predominantly IgG3 response [17, 18], while MSP-1 variants tend to illicit an IgG1-biased or mixed IgG1/IgG3 response [18]. CSP was expressed in a wheat germ cell-free system, and merozoite antigens were expressed in *Escherichia coli*. All antigens were based on the 3D7 allelic variant of *P. falciparum*.

### Detection of IgG and IgG Subclasses by High-Throughput Enzyme-Linked Immunosorbent Assay (ELISA)

Detection of IgG in response to CSP was performed by ELISA, using a robotic liquid handling system (the Janus automated work station; Perkin Elmer), as described previously [24]. Detailed methods are provided in the Supplementary Materials. Detection of IgG subclasses 1–4 was first performed manually by an ELISA, using samples from a randomly selected subset of participant samples representing each of the included study sites (n = 34). Additional IgG1 and IgG3 assays were then performed on samples from all participants (n = 984), using the Janus automated work station, as described previously [24]. Detailed methods are provided in the Supplementary Materials.

### Detection of Antibody-Dependent C1q Fixation

Antibody-dependent C1q fixation against recombinantly expressed MSP-2 (3D7) was determined by ELISA in all samples (n = 984) according to methods previously described [11]. Detailed methods are provided in the Supplementary Materials.

### Detection of Opsonic Phagocytosis

Phagocytosis of opsonized whole merozoites by unstimulated THP-1 cells was measured in a subset of participant samples from Thai and Cambodian study sites (n = 643), where a high proportion of participants had a PC_½_ of ≥5 hours [5]. Opsonic-phagocytosis assays were performed according to methods previously described [14]. Detailed methods are provided in the Supplementary Materials.

### Statistical Analysis

For each participant’s series of parasite counts, the PC_½_ was estimated from the slope of the linear segment of the log parasitemia-time curve, using the WWARN Parasite Clearance Estimator [25]. The seroprevalence of anti-CSP IgG was used as a proxy for prior exposure and, thus, transmission between study sites, as previously described [9], and was used for descriptive purposes only. Correlations between measured continuous antibody levels were assessed by Spearman rho with bootstrapping, and associations between binary IgG subclass seropositivity were assessed using Pearson χ^2^ tests. Meta-analyses were performed to examine study site heterogeneity in the mean differences in PC_½_ between seropositive and seronegative individuals, separately for each seropositivity measure. Forest plots and the *I*^*2*^ statistic were used to assess heterogeneity in the mean difference across study sites. If study site heterogeneity in the mean differences was negligible for an antigen, regression analyses were performed on data pooled across study sites. Where no participant from a particular study site was classified as seropositive for a subclass response, then that study site was omitted from the meta-analysis. Linear regression was performed on the primary outcome, PC_½_ (in hours), with a binary antibody measure (seropositive or seronegative) as the exposure, as well as transformed continuous antibody measures (log_2_ OD + 1) and continuous relative phagocytosis indexes (RPIs; Supplementary Table 2). To assess the effect of seropositivity on the binary treatment efficacy measures of a having a PC_½_ of ≥5 hours (yes or no) and having parasitemia on day 3 (yes or no), logistic regression was performed with a binary antibody measure (seropositive or seronegative) as the exposure, as well as transformed continuous antibody measures (log_2_ OD + 1) and continuous RPI (Supplementary Table 2). To assess effect modification by the presence of artemisinin-resistant *P. falciparum* strains, we included an interaction term for *kelch13* genotype (ie, having a validated resistance-associated single-nucleotide polymorphism or having a wild-type genotype) and compared models with and those without the interaction term, using a likelihood ratio test. All analyses were adjusted for age in years (mean centered) and artesunate dosage (2 or 4 mg/kg), and clustered standard errors were calculated to account for correlation between observations from the same study site. To estimate the total effect of immunity to blood-stage parasites, models were not adjusted for anti-CSP IgG because these antibodies are not independently associated with parasite clearance; rather, their association is mediated through blood-stage parasite immunity. All data were analyzed using Stata14 (StataCorp, College Station, TX).

## RESULTS

### Study Population

Participants were recruited from 11 Southeast Asian study sites, including sites where artemisinin resistance in *P. falciparum* has been confirmed. As reported previously, median PC_½_ values were greatest in western Cambodian (5.60 hours in Pursat and 6.10 hours in Pailin) and Thai (6.95 hours in Srisaket and 5.30 hours in Ranong) study sites, where the proportion of participants with parasites possessing *kelch13* mutations (range, 65%–83%) and the proportion of participants who were parasitemic at day 3 (range, 60%–73%) were also greatest (Table 1).

### Prevalence of IgG Subclasses and Functional Antibodies Varies by Study Site

Levels of IgG1 and IgG3 specific for the merozoite antigens EBA-175 RIII-V, MSP-2, and MSP-142 were determined in all participants’ samples (n = 984) after negligible IgG2 and IgG4 were detected in a random subset of samples (n = 34; Supplementary Figure 1). Overall, IgG3 was the predominant subclass, with higher seroprevalences than for IgG1 (EBA-175 RIII-V, 36% vs 5% [*P* < .001]; MSP-2, 27% vs 5% [*P* = .002], and MSP-142, 41% vs 35% [*P* = .01]). The seroprevalence of antibodies capable of fixing C1q in response to recombinantly expressed MSP-2 was 18.90% in the entire cohort, and the seroprevalence of antibodies mediating opsonic phagocytosis of free merozoites (3D7) was 92% in a subset of participants (n = 643) from the Thai and Cambodian study sites where the slow-clearing phenotype was prevalent. Weak-to-moderate positive correlations were found between IgG1 and IgG3 responses toward the same antigen and across antigens (Spearman ρ range, 0.16–0.49 [*P* < .001]; Supplementary Table 1). IgG1 and IgG3 responses were similarly correlated for C1q fixation between subclasses (Spearman ρ range, 0.16–0.25; *P* < .001), and IgG3 responses to all 3 antigens were more positively correlated with opsonic phagocytosis (Spearman ρ range, 0.24–0.42; *P* ≤ .001) than IgG1 responses (Spearman ρ range, 0.01–0.33 [*P* range, <.001–.76]; Supplementary Table 1).

**Figure 1.**
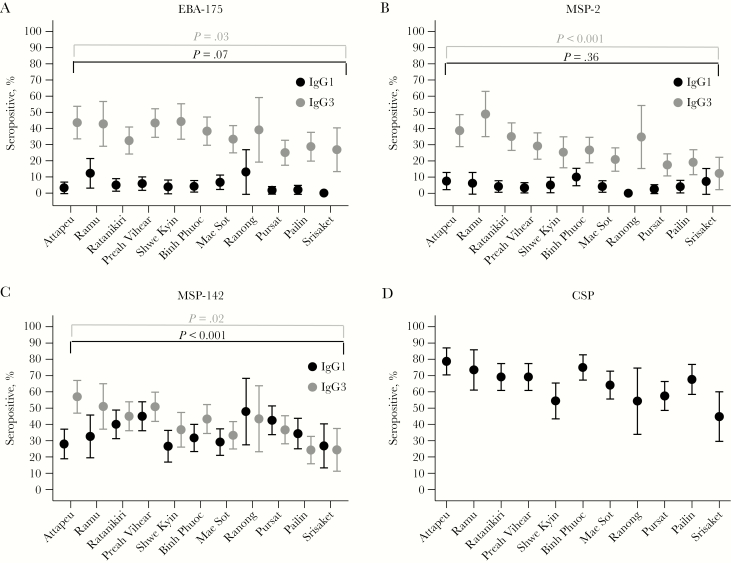
Seroprevalences, with 95% confidence intervals, of erythrocyte-binding antigen (EBA-175; *A*), merozoite surface protein 2 (MSP-2; *B*), and MSP-142 (*C*) immunoglobulin G1 (IgG1) and IgG3 and circumsporozoite protein (CSP) IgG across study sites. Seropositivity was defined as an OD greater than or equal to the mean value + 2 SDs for a panel of unexposed Melbourne donors. Study sites are ordered from fastest to slowest median parasite clearance half-life (in hours).

**Figure 2.**
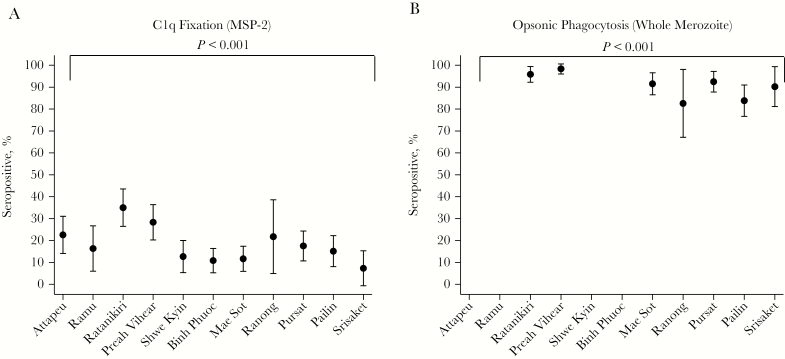
Seroprevalences, with 95% confidence intervals, for C1q fixation on recombinant merozoite surface protein 2 (MSP-2; *A*) and opsonic phagocytosis of whole merozoites (3D7; *B*) across study sites. Seropositivity was defined as an OD greater than or equal to the mean value + 2 SDs for a panel of unexposed Melbourne donors for C1q fixation. Opsonic-phagocytosis seropositivity was defined as a relative phagocytosis index (%) greater than or equal to the mean value + 3 SDs for a panel of unexposed Melbourne donors. Study sites are ordered from fastest to slowest median parasite clearance half-life (in hours).

The seroprevalences (Figure 1) and levels (Supplementary Figure 2) of IgG1 and IgG3 for all 3 antigens varied across study sites. IgG3 seroprevalences and levels were lowest in study sites with the lowest levels of transmission (using CSP seropositivity as a surrogate of preexisting exposure; Figure 1) and the slowest median PC_½_ (Supplementary Figure 2). Seroprevalences (Figure 2) and levels of C1q-fixing antibodies and opsonic phagocytosis of whole merozoites (Supplementary Figure 3) also varied by study site and were lowest in study sites with the lowest PC_½_ estimates.

### Cytophilic Antibody Measures Are Associated With Parasite Clearance Measures

To assess the association between IgG subclasses, their functional attributes, and measures of parasite clearance, we first assessed the heterogeneity in this association across study sites. Low-to-moderate heterogeneity in the mean difference in PC_½_ between seropositive and seronegative individuals between study sites was observed (median *I*^*2*^, 16.8% [range, 0%–63.7%]; Supplementary Figures 4–7). Potential clustering by study site was incorporated in multivariate regression analyses, which aimed to quantify the association between seropositivity and measurements of parasite clearance following artesunate treatment (ie, PC_½_, having a PC_½_ of ≥5 hours, and having parasitemia on day 3 following treatment), adjusting for age and artesunate-monotherapy dosage. IgG1 seropositivity was not associated with the PC_½_ after artesunate treatment, whereas IgG3 seropositivity was associated with a faster PC_½_ (mean difference in PC_½_ among seropositive individuals, −0.64 hours [95% confidence interval [CI], −.98 to −.31; *P* = .002] for EBA-175 RIII-V, −0.51 hours [95% CI, −.96 to −.05; *P* = .03] for MSP-2, and −0.47 hours [95% CI, −.88 to −.05; *P* = .03] for MSP-142; Table 2). Similarly, seropositivity for IgG1 was not associated with secondary treatment efficacy measures, whereas IgG3 responses were associated with a ≥35% reduced odds of having a PC_½_ of ≥5 hours and having parasitemia on day 3 after treatment (Table 2). C1q fixation in response to recombinantly expressed MSP-2 was associated with a significantly faster PC_½_ (mean difference in PC_½_ among seropositive individuals, −0.45 hours [95% CI, −.80 to −.09]; *P* = .02) and with a reduction in the odds of having a PC_½_ of ≥5 hours and having parasitemia on day 3 after treatment of 52% and 51%, respectively (Table 2). Similarly, opsonic phagocytosis of free merozoites was associated with a reduced PC_½_ (mean difference PC_½_ among seropositive individuals, −1.16 hours [95% CI, −2.17 and −.14]; *P* = .03) and with a reduction in the odds of having a PC_½_ of ≥5 hours and having parasitemia on day 3 after treatment of 66% and 48%, respectively (Table 2). No evidence was found for effect modification by the *kelch13* genotype (likelihood ratio *P* value range, .23–.99).

**Table 2.** Association Between Antibody Seroprevalence and Artemisinin Resistance Outcomes

	PC_½_, h			PC_½_ ≥ 5 h		Parasitemia at d 3	
Antibody	Mean Value, Reference Group^a^	Mean Difference^b^ (95% CI)	*P*	OR^b^ (95% CI)	*P*	OR^b^ (95% CI)	*P*
EBA-175 IgG1	3.72	−0.30 (−1.15–.55)	.46	0.85 (.48–1.52)	.60	0.91 (.49–1.67)	.75
EBA-175 IgG3	3.96	−0.64 (−.98 to −.31)	.002	0.57 (.41–.78)	<.001	0.62 (.46–.83)	.002
MSP-2 IgG1	3.72	−0.32 (−1.28–.64)	.48	0.81 (.39–1.68)	.57	0.92 (.52–1.64)	.79
MSP-2 IgG3	3.87	−0.51 (−.96 to −.05)	.03	0.65 (.39–1.06)	.09	0.58 (.34–.99)	.05
MSP-142 IgG1	3.66	0.13 (−.21–.47)	.42	1.12 (.85–1.46)	.43	1.14 (.86–1.52)	.35
MSP-142 IgG3	3.92	−0.47 (−.88 to −.05)	.03	0.60 (.44–.81)	.001	0.65 (.51–.81)	<.001
C1q fixation	3.80	−0.45(−.80 to −.09)	.02	0.48 (.36–.65)	<.001	0.49 (.37–.66)	<.001
Phagocytosis^c^	5.22	−1.16 (−2.17 to −.14)	.03	0.34 (.14–.85)	.02	0.52 (.29–.93)	.03

Estimates were determined by multivariate linear and logistic regression, adjusted for age and artesunate monotherapy dosage and robust standard errors for study site clustering.

Abbreviations: CI, confidence interval; EBA, erythrocyte-binding antigen; IgG, immunoglobulin G; MSP, merozoite surface protein; OR, odds ratio; PC_½_, parasite clearance half-life.

^a^Data are for seronegative individuals (average age, 26 years) who received 2 mg/kg artesunate monotherapy.

^b^Data are adjusted for age and artesunate monotherapy dosage and robust standard errors for study site clustering.

^c^Data are for a subset of Thai and Cambodian study sites (n = 643).

## DISCUSSION

In this multinational assessment of artesunate treatment efficacy, the proportion of IgG1-and IgG3-seropositive individuals varied significantly according to study site. The proportion of IgG3-seropositive individuals was lowest in the study sites with the slowest PC_½_ estimates. These were also the sites with the highest levels of artemisinin-resistant *P. falciparum.* Seroprevalences of IgG3, complement fixation, and opsonic phagocytosis of merozoites were associated with a faster PC_½_, a reduced odds of having a PC_½_ of ≥5 hours, and having parasitemia 3 days after starting artesunate treatment. The cytophilic IgG subclasses and associated functions that target merozoites are associated with parasite clearance measures used to assess the therapeutic efficacy of artemisinins and may be a sensitive surrogate of an augmented clearance capacity of infected erythrocytes, presumably by the spleen.

Both IgG1 and IgG3 antibodies have been shown previously to be produced at higher levels in response to *P. falciparum* merozoite targets, compared with IgG2 and IgG4, and are associated with protection from high-density parasitemia and clinical symptoms [16–19]. Consistent with this, IgG1 and IgG3 antibodies were the predominant IgG subclasses in response to the 3 merozoite antigens assessed in this cohort. Cytophilic IgG levels and seroprevalences varied by study site, and significant variations were observed for IgG3, C1q fixation, and opsonic-phagocytosis seroprevalence (*P* range, <.001–.03). Additionally, higher IgG3 level, and a higher proportion of IgG3-seropositive participants were found in study sites with the highest transmission (as estimated by seropositivity to CSP). These findings are consistent with evidence of a polarization toward a predominantly IgG3 response (and away from IgG1, IgG2, and IgG4 responses) with increasing transmission intensity, which has been limited to subnational studies in Africa and the Pacific [18, 21–23]

We found that IgG3 but not IgG1 specific for relatively conserved merozoite antigens was associated with significantly faster PC_½_ during treatment with artesunate. Distinct structural differences in the Fc portion and affinity for Fc-receptor binding on effector cells between the IgG1 and IgG3 subclasses results in differential ability to mediate functions such as phagocytosis by monocytes following opsonization, as well as complement fixation [20]. These differences in Fc-receptor affinity and binding may explain why IgG3 but not IgG1 seropositivity was associated with a faster PC_½_ when compared to findings for seronegative individuals. The seroprevalence of C1q-fixing antibodies in response to recombinant MSP-2 (a major target of merozoite C1q-fixing antibodies that is strongly correlated with C1q fixation against the whole merozoite [11, 26]) was associated with a faster PC_½_ and with secondary treatment efficacy measures, despite a low-to-moderate seroprevalence across study sites relative to other included antibody measures. The observed proportion of seropositive participants is considerably lower than in recent investigations of complement-fixing antibodies in a cohort study performed in a comparatively higher malaria-transmission setting of Papua New Guinea [26]. This is likely due to generally low levels of cytophilic IgG, compared with those detected in the Papua New Guinean cohort (IgG1 and IgG3 seroprevalence range, 40%–70%) [17, 18].

Antibody and complement interactions, opsonic phagocytosis of merozoites, and antibody inhibition of merozoite invasion all occur very rapidly, within minutes of egress [11, 14]. C1q fixation provides the most potent antibody-mediated merozoite invasion–inhibitory activity and is strongly associated with reductions in parasite density [11, 26], and its potent activity is the likely explanation for the large magnitude of the effect on PC_½_ that we observed. We found that opsonic phagocytosis of whole merozoites was associated with PC_½_ estimates that were >1 hour faster and with a reduction in the odds of having a PC_½_ of ≥5 hours and parasitemia detected on day 3, compared with seronegative individuals. Direct clearance of merozoites by phagocytosis may reduce parasite multiplication rates and have broader immune implications, such as activation of monocytes and production of proinflammatory cytokines [14]. Collectively, these results indicate that merozoite cytophilic and functional antibodies are associated with accelerated parasite clearance measures. These antibodies may contribute to parasite clearance either directly, by enhanced merozoite clearance and reduction of parasite multiplication, and/or through effects such as antibody-dependent cellular inhibition, which involves the release of soluble inhibitors of parasite growth [27]. Since complement can influence phagocytosis [28], future studies investigating the combined effect of antibodies and complement on phagocytosis of merozoites may be informative. Additionally, other immune mechanisms may also be important and warrant investigation in future studies to fully understand the interactions between immunity and parasite clearance in therapeutic efficacy studies.

The reduction in the circulating parasitemia level during *P. falciparum* malaria reflects several discrete processes. Changes in parasite densities in the hours immediately following treatment are largely independent of drug and reflect the balance between input of newly infected erythrocytes from deep vascular schizogony and sequestration of trophozoite-infected erythrocytes. So, after a variable lag phase, the parasitemia level declines in a log-linear manner (measured by the PC_½_). It is this measure that best reflects the ring-stage parasite killing effect of artemisinin drugs. Thereafter, in the absence of artemisinin resistance, input of newly infected erythrocytes (which will be reduced with effective antimerozoite immunity) contributes little to estimates of parasite clearance (ie, the PC_½_), although it can affect the parasite clearance time [29, 30]. During treatment of artemisinin-resistant *P. falciparum* infection, this rapid elimination of the ring stages is lost, and surviving parasites no longer susceptible to artemisinins will continue to mature and sequester, eventually giving rise to merozoites [30]. Antibodies may facilitate merozoite clearance through phagocytosis by effector cells [14], as well as through lysis or invasion inhibition by complement-dependent pathways, contributing to rates of parasite multiplication [11]. These mechanisms will also target other life cycle stages relevant to measures of parasite clearance. In this study, the 3 measures of parasite clearance were closely correlated, and the greatest effects on these measures in relation to transmission intensity was on opsonization and phagocytosis of merozoites. This suggests that antibody opsonization of merozoites is a sensitive surrogate of the augmented clearance capacity of infected erythrocytes, presumably by the spleen. *P. falciparum* ring stages no longer susceptible to artemisinins will go undamaged by treatment, allowing them to bypass rapid clearance in the spleen by mechanisms such as “pitting” and continue to mature [29]. However, in immune individuals, phagocytosis of artemisinin-resistant and, therefore, undamaged infected erythrocytes in the spleen may accelerate measures of parasite clearance. Previous small, single-site studies have shown that anti-VSA responses are associated with the PC_½_, using autologous parasite isolates [31, 32], and we have also previously shown in this multinational cohort that seropositivity for IgG to the surface of erythrocytes infected with laboratory strains of *P. falciparum* is associated with a faster PC_½_ [9]. However, VSAs vary across parasite populations. Therefore, to determine how different subclasses and their associated functions influence parasite clearance, we investigated these mechanisms in response to merozoite antigens because they are relatively conserved across study sites.

Other factors may also be important in understanding the role of immunity in parasite clearance, such as the parasite stage at the time of treatment initiation and the presence of *kelch13* mutant strains. Recent evidence from this cohort suggests that parasite stage at the time of treatment initiation moderately confounds the PC_½_ calculation but only in patients with *kelch13* mutants [7]; patients infected with a *kelch*13 mutant strain are more likely to have a PC_½_ of <5 hours when presenting with predominantly late as compared to early ring stages [7]. Antimerozoite immunity will act on the next egress/invasion event, which will occur earlier in those presenting with late ring stages. Other interactions observed between immunity and parasite clearance in this cohort were the differential effects of immunity to *kelch13* mutant and wild-type parasites on the PC_½_, whereby antimerozoite immunity had the greatest impact on the PC_½_ among individuals harboring *kelch13* mutants [9]. This interaction was not observed in analyses of IgG subclasses or functional antibodies, which may be due to differences in the detection sensitivity and relative seroprevalence of total versus cytophilic IgG. However, concordant with our previous findings, we observed some of the largest magnitudes of effect on the PC_½_ between IgG3-seropositive and IgG3-seronegative individuals in the study sites with the highest mean PC_½_ [9]. These observations highlight the complex contribution of host and parasite factors to measures of artemisinin treatment efficacy, which require inclusion in future assessments of artemisinin resistance phenotypes and emerging artemisinin resistance.

Detailed understanding of the effects of antibody functions that contribute to parasite clearance facilitates accurate estimation of within-host parasite dynamics and continued monitoring of the therapeutic efficacy of artemisinin treatments. We have shown that acquired IgG3 responses and antibody-mediated opsonic phagocytosis are associated with parasite clearance and other measures currently used to assess resistance to the artemisinins, as well as with the efficacy of antimalarial treatments.

## SUPPLEMENTARY DATA

Supplementary materials are available at *The Journal of Infectious Diseases* online. Consisting of data provided by the authors to benefit the reader, the posted materials are not copyedited and are the sole responsibility of the authors, so questions or comments should be addressed to the corresponding author.

jiz247_suppl_Supplementary_Figure_1

jiz247_suppl_Supplementary_Figure_2

jiz247_suppl_Supplementary_Figure_3

jiz247_suppl_Supplementary_Figure_4

jiz247_suppl_Supplementary_Figure_5

jiz247_suppl_Supplementary_Figure_6

jiz247_suppl_Supplementary_Figure_7

jiz247_suppl_Supplementary_Methodology

jiz247_suppl_Supplementary_Table_1

jiz247_suppl_Supplementary_Table_2

jiz247_suppl_Supplementary_Table_3

## Notes

***Acknowledgments.*** We thank all study participants and staff involved in the coordination of the TRAC study; Andrew Guy and Jack Richards, for experimental assistance; Robin Anders, for providing recombinant MSP-2; Carole Long, for providing recombinant MSP-142 antigens; AmrepFlow; and the Australian Red Cross Blood service.

E. A. A., F. N., J. A. S., N. J. W., and F. J. I. F. designed the research protocol; K. O., R. A., R. P., G. F., A. M. D., N. P. D., M. D., R. M. F., P. L., C. A., S. P., T. T. H., Y. H., M. M., M. A. F., F. N., J. A. S., N. J. W., and F. J. I. F. performed the research; L. R., J. G. B., and F. J. I. F. provided reagents/analytical tools; and K. O., S. Z., J. A. S., and F. J. I. F. wrote the manuscript.

***Disclaimer.*** The funders had no role in study design, data collection and analysis, decision to publish, or preparation of the manuscript.

***Financial support.*** This work was supported by the National Health and Medical Research Council of Australia (project grant 1060785 [to F. J. I. F., J. A. S., and F. N.], program grant APP637406 [to J. G. B.], training fellowship 637396 [to F. J. I. F.], and senior research fellowships APP1077636 [to J. G. B.] and 1104975 [to J. A. S.]); the Australian Centre for Research Excellence in Malaria Elimination (1134989); the Australian Research Council (Future Fellowships FT130101122 [to F. J. I. F.] and FT0992317 [to J. G. B.]); the Ramaciotti Foundation (Establishment Grant 3245/2011); the Ian Potter Foundation (grant to F. J. I. F.); the Australian Commonwealth Government (Australian Government Research Training Program Scholarship to K. O.); the Victorian State Government (Operational Infrastructure Support grant); the United Kingdom Department for International Development, with additional support from the Worldwide Antimalarial Resistance Network, and the Intramural Research Program of the National Institute of Allergy and Infectious Diseases, National Institutes of Health; and the Wellcome Trust of Great Britain (support to the Wellcome Trust Mahidol Oxford Tropical Medicine Research Programme).

***Potential conflicts of interest.*** All authors: No reported conflicts. All authors have submitted the ICMJE Form for Disclosure of Potential Conflicts of Interest. Conflicts that the editors consider relevant to the content of the manuscript have been disclosed.
