# Supplementary material for: Contribution of Functional Antimalarial Immunity to Measures of Parasite Clearance in Therapeutic Efficacy Studies of Artemisinin Derivatives
Source: J Infect Dis. 2019 May 10;220(7):1178–87. doi: 10.1093/infdis/jiz247 (PMC6735958; doi:10.1093/infdis/jiz247)
Supplement: jiz247_suppl_Supplementary_Figure_1 [file jiz247_suppl_supplementary_figure_1.docx]

**
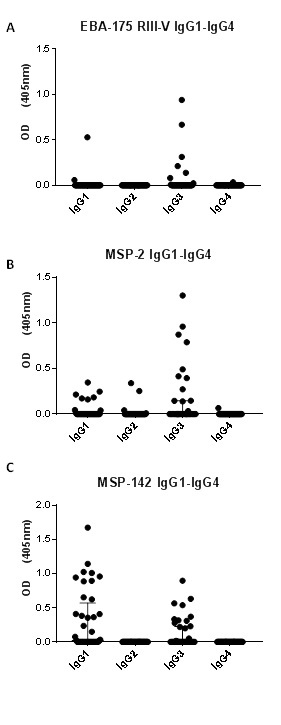
**

**Supplementary Figure 1:** Median (IQR) IgG1 – IgG4 levels (log_2_OD 405nm + 1) in response to merozoite antigens EBA-175 RIII-V (A), MSP-2 (B) and MSP142 (C) in a subset of participants (n = 34).
