# Supplementary material for: Contribution of Functional Antimalarial Immunity to Measures of Parasite Clearance in Therapeutic Efficacy Studies of Artemisinin Derivatives
Source: J Infect Dis. 2019 May 10;220(7):1178–87. doi: 10.1093/infdis/jiz247 (PMC6735958; doi:10.1093/infdis/jiz247)
Supplement: jiz247_suppl_Supplementary_Figure_2 [file jiz247_suppl_supplementary_figure_2.docx]

**
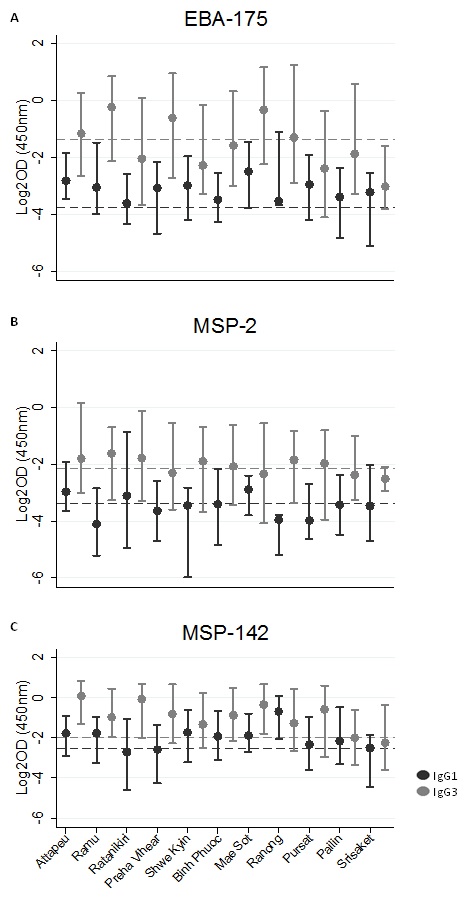
Supplementary Figure 2:** Median (IQR) IgG1 and IgG3 levels (log_2_OD 450nm) for EBA-175 (A), MSP-2 (B) and MSP-142 (C). Dashed lines are seropositivity cut-offs for each subclass.
