# Supplementary material for: Contribution of Functional Antimalarial Immunity to Measures of Parasite Clearance in Therapeutic Efficacy Studies of Artemisinin Derivatives
Source: J Infect Dis. 2019 May 10;220(7):1178–87. doi: 10.1093/infdis/jiz247 (PMC6735958; doi:10.1093/infdis/jiz247)
Supplement: jiz247_suppl_Supplementary_Figure_3 [file jiz247_suppl_supplementary_figure_3.docx]

**Supplementary Figure 3:** Median (IQR) C1q fixation (log_2_OD 450nm) (A) and opsonic phagocytosis levels (% Phagocytosis Index) (B) in response to whole merozoites. Dashed lines are seropositivity cut-off.

**B**

**A**
