# Supplementary material for: Contribution of Functional Antimalarial Immunity to Measures of Parasite Clearance in Therapeutic Efficacy Studies of Artemisinin Derivatives
Source: J Infect Dis. 2019 May 10;220(7):1178–87. doi: 10.1093/infdis/jiz247 (PMC6735958; doi:10.1093/infdis/jiz247)
Supplement: jiz247_suppl_Supplementary_Figure_7 [file jiz247_suppl_supplementary_figure_7.docx]

**Supplementary Figure 7: Forest plot of the effect of C1q fixation (A) and opsonic phagocytosis (B) seropositivity on PC½ (hours)** Meta-analyses were performed for each antigen response to determine the effect of seropositivity on PC½ (hours) between study sites. Values below 0 indicate faster PC½ in seropositive participants, and values above 0 indicate slower PC½ in seropositive participants. Studies sites are ordered from fastest to slowest median PC½.

**A**

**B**
