# Supplementary material for: Contribution of Functional Antimalarial Immunity to Measures of Parasite Clearance in Therapeutic Efficacy Studies of Artemisinin Derivatives
Source: J Infect Dis. 2019 May 10;220(7):1178–87. doi: 10.1093/infdis/jiz247 (PMC6735958; doi:10.1093/infdis/jiz247)
Supplement: jiz247_suppl_Supplementary_Methodology [file jiz247_suppl_supplementary_methodology.docx]

**Supplementary Material**

**Detailed methodology**

**Detection of IgG and IgG subclasses by high-throughput ELISA**

Detection of IgG in response to CSP was carried out using a robotic liquid handling system as described previously (JANUS automated work station, Perkin Elmer). Spectraplates were coated with 0.5µg/mL antigen overnight at 4°C. Plates were blocked for 2 hours at room temperature with 1% casein, and then incubated with patient sera at a concentration of 1:500 diluted in 0.1% casein PBS at room temperature for 2 hours. Goat anti-human HRP-conjugated antibody was added at a concentration of 1:1000 for one hour at room temperature. ABTS substrate was added to each well and covered for 20 minutes at room temperature, then stopped using 1% SDS, and read in a spectrophotometer at 405nm. IgG subclass ELISAs (IgG1 - IgG4) in responses to *P. falciparum* antigens EBA-175, MSP-2 and MSP1-42 were first performed in a randomly selected subset of participant samples from each of the included study sites (n = 34). Spectraplates were coated with 0.5 µg/mL antigen (MSP-2, EBA-175 RIII-V, MSP1-42) overnight at 4°C. Plates were blocked for 2 hours at room temperature with 10% skim milk, and then incubated with patient sera at a concentration of 1:1000 diluted in 5% skim milk PBS at room temperature for 2 hours. Mouse anti-human IgG1 – IgG4 antibody was added at a concentration of 1:1000 for one hour at room temperature, followed by goat anti-human HRP-conjugated antibody incubated for an hour at room temperature. ABTS substrate was added to each well and covered for 20 minutes at room temperature, then stopped using 1% SDS, and read in a spectrophotometer at 405nm. Additional IgG1 and IgG3 assays were performed in all participants (n = 984) using a robotic liquid handling system (JANUS automated work station, Perkin Elmer) following the same protocol, however, to improve sensitivity TMB substrate was added to each well and covered for 20 minutes at room temperature, then stopped using 1M sulphuric acid, and read in a spectrophotometer at 450nm. Wells containing no test sera were used to deduct background reactivity from each sample, and a seropositivity cut-off point was set at an OD above the mean + 2SDs of a panel of Melbourne donors. Values generated across multiple plates were standardised through calculating a conversion factor as follows; firstly, the mean of all blank wells within that plate (wells containing no sera samples) is subtracted from the OD reading of each sample to account for background reactivity. Following this, the mean of each positive control used across each plate is calculated. Positive controls were then divided by the mean of that positive control across all plates. Finally, the mean of these values on a single plate is used as a conversion factor for that plate. A normalized value is created for each sample by dividing it by the conversion factor of that reaction plate.

**Detection of C1q fixing antibodies by ELISA**

Spectraplates were coated with recombinant MSP-2 at 0.5 µg/mL in PBS overnight, then blocked with 1% casein in PBS for 2 hours at 37°C. Plates were washed then incubated with patient sera at a concentration of 1/250 diluted in 0.1% casein PBS for 2 hours at 37°C. After washing, plates were incubated with purified human C1q (10µg/mL) for 30 minutes, before washing and the addition of rabbit anti-C1q antibodies at a concentration of 1/2000 for 1 hour at 37°C, then washed and incubated with goat anti-rabbit antibodies at a concentration of 1/2000 for 1 hour at 37°C. TMB substrate was added to each well and covered for 30 minutes at room temperature, then stopped using 1M sulphuric acid, and read in a spectrophotometer at 450nm. The mean of wells containing no test sera on each plate were used to deduct background reactivity from each sample, and a seropositivity cut-off point was set at an OD above the mean + 2SDs of a panel of Melbourne donors. Values generated across multiple plates were standardised through calculating a conversion factor as follows; firstly, the mean of all blank wells within that plate (wells containing no sera samples) is subtracted from the OD reading of each sample to account for background reactivity. Following this, the mean of each positive control used across each plate is calculated. Positive controls were then divided by the mean of that positive control across all plates. Finally, the mean of these values on a single plate is used as a conversion factor for that plate. A normalized value is created for each sample by dividing it by the conversion factor of that reaction plate.

**Detection of Opsonic Phagocytosis**

Purified merozoites pre-stained with Et-Br (1µg/mL) at a volume of 30µL were incubated for one hour with 3.3µL of neat heat-inactivated patient sera. Plates were then washed three times with incomplete RPMI and centrifuged at 300 x g for 4 minutes before being resuspended in 150µL of THP-1 culture medium. Opsonized merozoites were transferred into three replicates by transferring 50µL of the parasite suspension into 100µL of THP-1 cell culture concentrated at 5 x 10⁵ cells/mL and incubated for 10 minutes at 37°C. Following incubation, plates were washed three times by cold FACS buffer (PBS, 0.5-1%, 5-10% Fetal Bovine Serum (FBS), 0.1% NaN3 sodium azide) and centrifuged at 300 x g for 4 minutes at 4°C before being fixed with cold 2% paraformaldehyde in PBS and analysed using a BD FACS Canto II flow cytometer (BD Biosciences: Franklin Lakes, NJ). A Relative Phagocytosis Index (RPI %) was calculated by comparing the proportion of THP-1 that had taken up stained merozoites with a standard curve generated from positive controls. Merozoites incubated without sera were used as un-opsonized controls, and positive controls included highly reactive sera pooled from exposed individuals. Samples with an RPI % exceeding the mean + 3 SDs of a panel of Melbourne controls were considered seropositive.
