# Supplementary material for: Contribution of Functional Antimalarial Immunity to Measures of Parasite Clearance in Therapeutic Efficacy Studies of Artemisinin Derivatives
Source: J Infect Dis. 2019 May 10;220(7):1178–87. doi: 10.1093/infdis/jiz247 (PMC6735958; doi:10.1093/infdis/jiz247)
Supplement: jiz247_suppl_Supplementary_Table_1 [file jiz247_suppl_supplementary_table_1.docx]

|  | | | | | | | | |  |
| --- | --- | --- | --- | --- | --- | --- | --- | --- | --- |
| **Supplementary Table 1: Spearman’s**  ρ **(95%CI), p value, for correlations between measured responses** | | | | | | | | |  |
|  | **EBA-175 IgG1** | **EBA-175 IgG3** | **MSP-2 IgG1** | **MSP-2 IgG3** | **MSP-142 IgG1** | **MSP-142 IgG3** | **C1q**  **(MSP-2)** | **Opsonic Phagocytosis** | |
| **EBA-175 IgG1** | 1 |  |  |  |  |  |  |  | |
| **EBA-175 IgG3** | 0.46 (0.40, 0.51), *<0.001* | 1 |  |  |  |  |  |  | |
| **MSP-2 IgG1** | 0.30 (0.23, 0.37), *<0.001* | 0.22 (0.16, 0.29), *<0.001* | 1 |  |  |  |  |  | |
| **MSP-2 IgG3** | 0.16 (0.10, 0.22), *<0.001* | 0.26 (0.21, 0.32), *<0.001* | 0.11 (0.05, 0.18), *<0.001* | 1 |  |  |  |  | |
| **MSP-142 IgG1** | 0.28 (0.22, 0.34), *<0.001* | 0.23 (0.17, 0.28), *<0.001* | 0.27 (0.21, 0.33), *<0.001* | 0.07 (0.01, 0.13), *0.03* | 1 |  |  |  | |
| **MSP-142 IgG3** | 0.33 (0.28, 0.39), *<0.001* | 0.49 (0.44, 0.55), *<0.001* | 0.16 (0.10, 0.22), *<0.001* | 0.25 (0.19, 0.31), *<0.001*) | 0.40 (0.35, 0.46), *<0.001* | 1 |  |  | |
| **C1q**  **(MSP-2)** | 0.19 (0.13, 0.25), *<0.001* | 0.16 (0.09, 0.23), *<0.001* | 0.15 (0.09, 0.22), *<0.001* | 0.20 (0.13, 0.26), *<0.001* | 0.25 (0.19, 0.31), *<0.001* | 0.24 (0.18, 0.30), *<0.001* | 1 |  | |
| **Opsonic Phagocytosis ^a^** | 0.19 (0.12, 0.27), *<0.001* | 0.30 (0.24, 0.37), *<0.001* | 0.01 (-0.06, 0.09), *0.76* | 0.24 (0.16, 0.31), *<0.001* | 0.33 (0.26, 0.40), *<0.001* | 0.42 (0.35, 0.48), *<0.001* | 0.19 (0.13, 0.27), *<0.001* | 1 | |
| **^a^** Opsonic phagocytosis completed in a subset of Thai and Cambodian study sites (n = 643) | | | | | | | | |  |
