# Supplementary material for: Contribution of Functional Antimalarial Immunity to Measures of Parasite Clearance in Therapeutic Efficacy Studies of Artemisinin Derivatives
Source: J Infect Dis. 2019 May 10;220(7):1178–87. doi: 10.1093/infdis/jiz247 (PMC6735958; doi:10.1093/infdis/jiz247)
Supplement: jiz247_suppl_Supplementary_Table_2 [file jiz247_suppl_supplementary_table_2.docx]

| **Supplementary Table 2: The association between continuous antibody measures (log_2_OD + 1) and artemisinin resistance outcomes** | | | | | | | |
| --- | --- | --- | --- | --- | --- | --- | --- |
| **Antibody** | **PC½ (hours)** | | | **PC½ ≥ 5 hours** | | **Parasitemia at day 3** | |
|  | **Mean PC½ (hours)**  **in reference group ^a^** | **Mean change ^b, c^ in PC½ (hours)**  **(95% CI)** | ***p*** | **OR ^c, d^ (95% CI)** | ***p*** | **OR ^c, e^ (95% CI)** | ***p*** |
| EBA-175 IgG1 | 3.71 | -0.27 (-1.11, 0.56) | *0.48* | 0.94 (0.52, 1.69) | *0.83* | 0.87 (0.42, 1.80) | *0.70* |
| EBA-175 IgG3 | 3.72 | -0.41 (-0.67, -0.16) | *0.005* | 0.68 (0.56, 0.83) | *<0.001* | 0.67 (0.49, 0.92) | *0.01* |
| MSP-2 IgG1 | 3.70 | -0.27 (-2.56, 2.01) | *0.80* | 0.60 (0.17, 2.20) | *0.45* | 0.67 (0.24, 1.89) | *0.45* |
| MSP-2 IgG3 | 3.73 | -0.57 (-1.07, -0.06) | *0.03* | 0.61 (0.35, 1.08) | *0.09* | 0.54 (0.29, 0.98) | *0.04* |
| MSP-142 IgG1 | 3.71 | 0.34 (-0.12, 0.79) | *0.13* | 1.24 (0.78, 1.99) | *0.36* | 1.27 (0.79, 2.04) | *0.32* |
| MSP-142 IgG3 | 3.74 | -0.41 (-0.83, 0.01) | *0.06* | 0.69 (0.44, 1.07) | *0.10* | 0.77 (0.52, 1.15) | *0.20* |
| C1q Fixation | 3.53 | -0.09 (-0.16, -0.01) | *0.03* | 0.87 (0.84, 0.92) | *<0.001* | 0.90 (0.86, 0.94) | *<0.001* |
| Phagocytosis ^f^ | 4.17 | -0.20 (-0.44, 0.04) | *0.09* | 0.83 (0.71, 0.96) | *0.01* | 0.85 (0.76, 0.95) | *0.004* |
| Estimates derived from multivariate linear and logistic regression, adjusted for age and artesunate monotherapy dosage and robust standard errors for study site clustering, ^a^ Mean PC½ in individuals of average age (26 years) given 2mg/kg artesunate monotherapy with geometric mean antibody level, ^b^ Mean change in PC½ for every 2-fold increase in antibody measure in individuals of average age (26 years) given 2mg/kg artesunate monotherapy, ^c^ Adjusted for age and artesunate monotherapy dosage, ^d^ Relative change in the odds of PC½ ≥ 5 hours for every two-fold increase in antibody measure, ^e^ Relative change in the odds of parasitemia at day 3 for every two-fold increase in antibody measure, ^f^ Opsonic phagocytosis completed in a subset of Thai and Cambodian study sites (n = 643) | | | | | | | |
