# Supplementary material for: Contribution of Functional Antimalarial Immunity to Measures of Parasite Clearance in Therapeutic Efficacy Studies of Artemisinin Derivatives
Source: J Infect Dis. 2019 May 10;220(7):1178–87. doi: 10.1093/infdis/jiz247 (PMC6735958; doi:10.1093/infdis/jiz247)
Supplement: jiz247_suppl_Supplementary_Table_3 [file jiz247_suppl_supplementary_table_3.docx]

| **Supplementary Table 3: The association between antibody seroprevalence and artemisinin resistance outcomes in participants included in opsonic phagocytosis analyses (n = 643)** | | | | | | | |
| --- | --- | --- | --- | --- | --- | --- | --- |
| **Antibody** | **PC½ (hours)** | | | **PC½ ≥ 5 hours** | | **Parasitemia at day 3** | |
|  | **Mean PC½ (hours)**  **in reference group ^a^** | **Mean difference ^b^ in PC½ (hours)**  **(95% CI)** | ***p*** | **OR ^b^ (95% CI)** | ***p*** | **OR ^b^ (95% CI)** | ***p*** |
| EBA-175 IgG1 | 4.13 | 0.09 (-0.81, 0.99) | *0.83* | 0.94 (0.56, 1.57) | *0.81* | 1.25 (0.58, 2.19) | *0.73* |
| EBA-175 IgG3 | 4.36 | -0.58 (-1.14, -0.02) | *0.04* | 0.58 (0.36, 0.92) | *0.02* | 0.64 (0.42, 0.86) | *0.03* |
| MSP-2 IgG1 | 4.14 | 0.10 (-1.16, 1.37) | *0.85* | 0.95 (0.54, 1.67) | *0.86* | 1.11 (0.71, 1.74) | *0.64* |
| MSP-2 IgG3 | 4.34 | -0.69 (-1.05, -0.33) | *0.002* | 0.54 (0.29, 1.01) | *0.05* | 0.44 (0.30, 0.66) | *<0.001* |
| MSP-142 IgG1 | 4.13 | 0.03 (-0.46, 0.52) | *0.90* | 0.96 (0.71, 1.29) | *0.77* | 1.03 (0.79, 1.34) | *0.83* |
| MSP-142 IgG3 | 4.31 | -0.38 (-0.84, 0.08) | *0.09* | 0.61 (0.42, 0.87) | *0.01* | 0.62 (0.46, 0.84) | *0.002* |
| C1q Fixation | 4.19 | -0.51 (-0.88, -0.13) | *0.01* | 0.42 (0.31, 0.57) | *<0.001* | 0.44 (0.33, 0.60) | *<0.001* |
| Phagocytosis | 5.22 | -1.45 (-2.37, -0.53) | *0.01* | 0.34 (0.14, 0.85) | *0.02* | 0.52 (0.29, 0.93) | *0.03* |
| ^a^ Mean PC½ in seronegative individuals of average age (26 years) given 2mg/kg artesunate monotherapy, ^b^ Adjusted for age and artesunate monotherapy dosage | | | | | | | |
